# Strengthening Healthcare through Academic and Industry Partnership Research

**DOI:** 10.3126/nje.v13i2.58243

**Published:** 2023-08-31

**Authors:** Brijesh Sathian, Edwin van Teijlingen, Padam Simkhada, Indrajit Banerjee, Hemanth Kumar Manikyam, Russell Kabir

**Affiliations:** 1 Geriatrics and long term care department, Rumailah Hospital, Hamad Medical Corporation, Doha, Qatar; 2 Centre for Midwifery & Women’s Health, Bournemouth University, Bournemouth, UK; 3 School of Human and Health Sciences, University of Huddersfield, Huddersfield, United Kingdom; 4 Sir Seewoosagur Ramgoolam Medical College, Belle Rive, Mauritius; 5 Faculty of Science, North East Frontier Technical University, Arunachal Pradesh, India; 6 School of Allied Health, Anglia Ruskin University, Essex, UK

##  

The foundation of advancement in healthcare has traditionally been research, which has sparked developments that helped improve patient outcomes, raise quality of life, and address current issues. The main source of innovation is the knowledge exchange between academic institutions and other economic actors, such as industry, the media, governmental agencies, and the general public [1]. As opposed to being considered a "third mission" activity, knowledge transfer, engagement, and impact are now at the core of what higher education institutions (HEIs) accomplish [2].

HEIs all over the world have greatly grown their business relationships in recent decades due to social pressure to improve their contributions to regional and national economic development. The synergy between academics and industry has become a powerful force in the United Kingdom (UK), encouraging a dynamic partnership that capitalizes on the distinct advantages of both sectors. Two decades ago the UK started its Knowledge Transfer Partnerships (KTP) programme, encourages collaboration between HEIs and industry. In 2023 there is up to £40 million for businesses to collaborate with a HEI and have a graduate through a KTP [3]. The UK's introduction of Industry Partnership Research Grants represented a crucial step in realizing the full potential of this partnership and opening the door to game-changing medical discoveries that will benefit patients and society at large. Currently the UK Research Partnership Investment Fund (UKRPIF) supports investment in HEIs, the latter must attract a further £2 from non-public sources (i.e. industry) for every £1 invested by the UKRPIF.

There are many examples available in the health and medical sector of successful academic-industry partnerships. For example, AstraZeneca and the University of Cambridge have teamed up to create new cancer therapies [4]. Whilst Novo Nordisk and the University of Oxford have teamed up to create new diabetic medicines [5]. The company GSK and Imperial College London are collaborating to help create fresh vaccinations against infectious diseases [6]. In terms of health research, GSK and Imperial College London have a long history of cooperation. The Engineered Medicines Laboratory (EML), which was created in 2016, is one of their most recent collaborations. The EML is a special partnership between the two organisations with the objective of creating revolutionary advancements in the identification, creation, and production of upcoming pharmaceuticals. Pfizer and University College London are working together to create novel therapies for rarer uncommon diseases [7]. These are only a few instances of the many academic-industry collaborations that are occurring in the UK's health research.

Across universities, the level and intensity of industry-university partnership varies. A study by Bertoletti and Johnes, analysing 164 universities in the UK, classified HEIs into two categories: (1) small ones with a weaker research orientation, and (2) larger research-intensive HEIs, with relatively high shares of staff in STEM (Science, Technology, Engineering, and Mathematics) disciplines [8]. The expansion of research-intensive universities and the creation of enabling economic environments for knowledge transfer should be the main priorities for policymakers. They finished by talking about the findings of policymaking implications. Bertoletti and Johnes argued that research-intensive universities are more likely to collaborate effectively with industry, policymakers should concentrate on fostering the growth of these HEIs. They suggested that lawmakers should concentrate on fostering economic environments that facilitate the transfer of information, such as by offering tax benefits to companies that partner with universities. However, smaller universities are perhaps more likely to come up with more novel small-scale local collaborations and hence solutions. For example, Bournemouth University in the south of England established clinical doctorates to enable local clinicians to do a PhD in their NHS (National Health Services) workplace [9]. This clinical doctorate takes a minimum of four years instead of the three-years standard PhD to allow clinicians to stay in the health services (i.e. industry) whilst completing their studies.

Encouraging academic and industrial cooperation in the UK with the goal of boosting innovation, knowledge exchange, and economic expansion. Both sectors seek to utilize their unique strengths through strategic alliances in order to tackle current issues, advance research and development, and improve the overall competitiveness of the country on a worldwide scale. The potential of collaboration, accelerating innovation, boosting patient-centered research, assuring ethical standards and transparency, and developing long-lasting partnerships are the most crucial factors that should be taken care of by both organizations.

Academic institutions contribute to fundamental research, intellectual rigour, and the pursuit of knowledge for the benefit of society to the UK healthcare scene. In contrast, industries have the technical know-how, support systems, and funding to turn research discoveries into workable solutions and marketable goods and services. The UK can bridge the gap between theoretical discoveries and practical applications by properly applying the combined power of these two organizations, accelerating the journey of medical advances from laboratories to the patient's bedside. The UK's collaboration between academia and industry has the potential to significantly quicken the speed of medical innovation. Research can be carried out with an emphasis on practical applicability by fusing academic rigour and curiosity with industry-driven pragmatism. This strategy makes ensuring that the information produced through these funds directly addresses the demands and difficulties faced by UK healthcare professionals, managers and patients. The commercial viability, market demands, and scalability of prospective solutions can also be greatly influenced by industry partners, resulting in the translation of ground-breaking research into advances in healthcare that are both accessible and affordable.

Grants supporting academic and industrial collaboration in the UK are highly valued since such grants can act as a spur to conduct patient-centered studies. Industry partners' active participation enables a deeper comprehension of patients’ requirements and preferences as well as the practical effects of prospective solutions. These awards encourage the development of novel solutions that are specifically tailored to meet the needs of the different patient populations in the UK by actively including patients in the research process. To ensure that research outputs directly and truly assist people who need it most, it is essential to place a strong emphasis on patient-centeredness.

While there is no denying the benefits of academic and industrial collaboration, it is crucial in the UK context to retain ethical standards and ensure transparency throughout the research process. The management of any conflicts of interest, protection of academic freedom, and integrity of the research are crucial. The UK can guarantee that the growth of scientific knowledge stays at the forefront, with the ultimate goal being the improvement of healthcare for the benefit of its population, by setting strict criteria and oversight procedures.

In the UK, the success of research grants depends on fostering enduring, long-term partnerships between academia and industry. The two sectors must foster an atmosphere of mutual respect, open communication, and trust in order to achieve this. Through collaborative seminars, forums between academia and industry, and shared governance models that are specifically designed to meet the requirements of the UK's healthcare system, these collaborations can be strengthened. The UK can boost the potential to change healthcare and promote a healthy nation by actively engaging in these connections and ensuring that both sides are equally dedicated to the research's success.

### Knowledge Exchange Framework

In the UK knowledge exchange outcomes now being measured annually for each university. This process is commonly known as KEF (Knowledge Exchange Framework). The KEF aims to allow universities to better understand and improve their own performance in knowledge exchange, as well as providing companies with more information to help them access HEI’s knowledge and expertise. The KEF is providing a league table with similar kinds of universities grouped together, based on how much research they do and in what subject areas. Their performance is then presented alongside the average performance of this group of similar HEIs. Both HEI’s of the UK based authors of this paper, Bournemouth University and the University of Huddersfield, are in KEF group E. Group E is defined as: large universities with broad discipline portfolio across both STEM and non-STEM generating excellent research across all disciplines; with a significant amount of research funded by government bodies/hospitals; 9.5% from industry; and a large proportion of part-time undergraduate students; and a small postgraduate population dominated by taught postgraduate courses.

### COVID-19 impact on academia and industry partnership

The epidemic has highlighted the value of industrial and academic cooperation in addressing urgent issues [10]. As an illustration, pharmaceutical corporations and academic researchers have collaborated to create novel COVID-19 vaccines and therapies. It became simpler for academics and industry to work together, often online so it did not matter where they where physically located. This was very much part of the shift to remote working during the pandemic. This has made the flow of ideas and information more open. The pandemic has also resulted in a rise in the usage of internet platforms and video conferencing as well as other digital tools for communication. These platforms have imprived rapidly since the start of COVID-19, and hence made it simpler for scholars and industry executives to collaborate on projects. The pandemic has brought innovation into greater spotlight, emphasizing its significance in solving problems. As a result, industry and academics are working together more closely as they both search for novel methods to solving challenges. AstraZeneca, BioNTech, Moderna, and Pfizer are now as well-known as brands as a result of the pandemic. But without exceptional and quick cooperation with academic researchers, supported by government funding, their life-saving vaccines would not have materialized.

### Final thoughts

Academic and Industry Partnership Research Grants have a huge potential to improve healthcare in the UK and elsewhere. This potent partnership between academia and business can hasten medical innovation, strengthen patient-centered research, and close the gap between theoretical understanding and real-world implementations. We can make sure that the quest of scientific knowledge continues to be at the forefront of healthcare developments by welcoming this partnership while respecting ethical norms and transparency. The UK's dedication to developing long-term alliances based on mutual respect and open dialogue will help create a climate in which ground-breaking research may flourish and be transformed into affordable healthcare solutions.
